# High-quality targeted temperature management combined with decompressive craniectomy in patients with poor-grade aneurysmal subarachnoid hemorrhage: a secondary analysis of a multicenter prospective study

**DOI:** 10.3389/fneur.2024.1483037

**Published:** 2025-01-06

**Authors:** Yang Liu, Bingsha Han, Yanru Li, Zhiqiang Ren, Yong Chen, Ming Zhang, Jiao Li, Jv Wang, Fan Yang, Mengyuan Xu, Jiaqi Zhang, Pengzhao Zhang, Tiancai Wang, Jinying Tian, Guang Feng

**Affiliations:** ^1^Department of Neurosurgery, Zhengzhou University People's Hospital, Henan Provincial People's Hospital, Zhengzhou, China; ^2^Department of Neurosurgical Intensive Care Unit, Henan Provincial People's Hospital, Zhengzhou, China; ^3^Department of Neurosurgery, Henan University People's Hospital, Henan Provincial People's Hospital, Zhengzhou, China; ^4^Graduate School of Xinxiang Medical University, Xinxiang, China; ^5^Department of Intensive Care Unit, Nanshi Hospital of Nanyang, Nanyang, China; ^6^Department of Intensive Care Unit, Sanmenxia Central Hospital, Sanmenxia, China

**Keywords:** aneurysmal subarachnoid hemorrhage, decompressive craniectomy, hypothermia, target temperature management, neurological outcome

## Abstract

**Background:**

The effect of targeted temperature management (TTM) combined with decompressive craniectomy (DC) on poor-grade aneurysmal subarachnoid hemorrhage (aSAH) has not been previously addressed in the literature. This study aims to investigate the therapeutic outcomes of the combination of TTM and DC in patients with poor-grade aSAH.

**Methods:**

This study represents a secondary analysis of the Multicenter Clinical Research on Targeted Temperature Management of Poor-grade Aneurysmal Subarachnoid Hemorrhage (High-Quality TTM for PaSAH), a multicenter prospective study conducted in China. The High-Quality TTM for PaSAH study enrolled patients aged 18 years and older who were transported to the intensive care units (ICU) of three tertiary care hospitals in China between April 2022 and April 2024. Among these patients, those who underwent DC were included in the present analysis. Patients were divided into two groups: the DC-alone group and the TTM combined with the DC (TTM-DC) group. The DC-alone group maintained normothermia. The TTM-DC group used automated devices with a temperature feedback system (TFS). TTM was initiated with core temperatures between 36°C-37°C immediately after diagnosing poor-grade aSAH, and concurrent emergency aneurysm repair. This was followed by a rapid induction to 34°C-35°C, maintained for a minimum of 72 h. Subsequently, a slow rewarming process reached 36°C-37°C, which was maintained for at least 48 h. Primary outcomes were evaluated using the Modified Rankin Scale (mRS) score at 3 months. Secondary outcomes included the Glasgow Coma Scale (GCS) at discharge, ICU stay duration, length of hospitalization, proportion of external ventricular drainage (EVD), mechanical ventilation time, tracheostomy, midline shift, hydrocephalus, and delayed cerebral ischemia (DCI) on the 7^th^ day. Safety outcomes comprised the incidence of pneumonia, myocardial infarction, stress hyperglycemia, thrombocytopenia, acute liver injury, hypokalemia, hypoproteinemia, and death at 90 days.

**Results:**

Of the 141 patients enrolled in the High-Quality TTM for PaSAH study, 43 (25 in the TTM-DC group and 18 in the DC-alone group) were eligible for this secondary analysis. The TTM-DC group had a higher proportion of favorable outcomes (mRS 0–3: 56% vs. 22%, aOR 5.97, 95%CI 0.96–52.2, *p* = 0.071). After propensity score matching, the TTM combined with DC improved favorable outcome at 3 months (mRS 0–3: 61% vs. 22%, OR 5.50, 95%CI 1.36–26.3, *p* = 0.022). In addition, the TTM-DC group increased GCS score at discharge compared with the DC-alone group (9 vs. 3, *β* 2.58, 95%CI 0.32–4.84, *p* = 0.032). The incidence of safety outcomes was not increased in the TTM-DC group.

**Conclusion:**

TTM combined with DC can improve clinical conditions at discharge and ameliorate short-term neurological outcomes in poor-grade aSAH patients. TTM should be considered one of the main treatments for poor-grade aSAH patients who underwent DC.

## 1 Introduction

Aneurysmal subarachnoid hemorrhage (aSAH) results from the abnormal bulging and rupture of an intracranial artery wall. The global annual incidence of aSAH ranges from 2 to 16 per 100,000 individuals, constituting 8% of all stroke cases (1). aSAH, as a common neurocritical disease in neurosurgery, remains the leading cause of death and disability among hemorrhagic strokes. A study involving 5 million Chinese adults revealed that aSAH patients represented 2% of stroke cases, particularly affecting females and individuals aged 50 and above (2). Patients with poor-grade aSAH (Hunt-Hess grade IV-V, modified Fisher (mFisher) grade 3–4, World Federation of Neurosurgical Societies (WFNS) grade IV-V) make up around 25 and 40% of aSAH cases, respectively, with a case fatality rate exceeding 50% and unfavorable clinical outcomes (3, 4). The economic burden imposed by poor-grade aSAH on society and families worldwide is substantial.

Early brain injury (EBI) includes diffuse cerebral edema from bleeding, microcirculatory alterations, oxidative and inflammatory cascade, and blood–brain-barrier breakdown, all of which contribute to neuronal damage (5, 6). A severe complication of poor-grade aSAH is an acute increase in ICP. The main causes of elevated ICP are hydrocephalus (30%), intracerebral hemorrhage (ICH), and early or delayed global cerebral edema (GCE), which account for 8–12% of cases (7, 8). Elevated ICP can cause reduced cerebral perfusion pressure and brainstem herniation. Refractory-raised ICP is associated with poor outcomes (9, 10). The management of increased ICP in poor-grade aSAH patients lacks specific guidelines, prompting the application of experiences from traumatic brain injury (TBI) guidelines (11, 12). However, aSAH is different from TBI in pathophysiology (13). Standard first-line treatments in treating high ICP in poor-grade aSAH include optimizing cerebral venous outflow, controlled hyperventilation, sedation with analgesia, cerebrospinal fluid diversion, and surgical removal of space-occupying intracranial hematoma. Second-tier treatment is osmotherapy, including mannitol, hypertonic saline, furosemide, and albumin. Third-tier treatments include therapeutic hypothermia (TH), barbiturate coma, and decompressive craniectomy (DC) (6). Major determinants of poor functional outcome and case fatality are EBI, rebleeding of the ruptured aneurysm, and delayed cerebral ischemia (DCI) (14).

Numerous prior studies have demonstrated that TH can regulate core temperature while also decreasing brain metabolism, reducing apoptosis in brain cells, alleviating brain tissue inflammation, and minimizing the formation of free radicals. Target temperature management (TTM) is an effective therapeutic approach for brain protection and encompasses TH, normothermia control, and fever control. The benefits of TTM in severe neurological diseases are becoming increasingly apparent, including in cases such as cardiac arrest, severe TBI, ischemic stroke, and neonatal ischemic brain injury (15–18). Nonetheless, there is a notable lack of research on TTM in poor-grade aSAH, and it remains unclear whether TTM combined with DC yields more favorable outcomes and lower mortality. To further clarify the effects of TTM combined with DC in poor-grade aSAH patients, we analyzed the data obtained from a multicenter prospective study and compared the outcomes of those who were treated with TTM combined with DC or DC alone.

## 2 Methods

### 2.1 Patient population

This study presents a secondary analysis of a multicenter, prospective, double-blind trial conducted by the Department of Intensive Care Unit (ICU) across three hospitals in China from April 2022 to April 2024. The trial is registered in the Chinese Clinical Trial Registry with the identifier ChiCTR2300075540. We included patients aged 18 to 80 years who were diagnosed with aSAH via Computed Tomography Angiography (CTA) or Digital Subtraction Angiography (DSA) within 24 h of symptom onset. Exclusion criteria for this trial encompassed systemic inflammatory response syndrome, sepsis, abnormal coagulation function, craniocerebral trauma, gastrointestinal bleeding, urinary bleeding, renal failure, as well as pregnant and lactating patients, as detailed in our previously published study. Patients were categorized into two groups: the DC-alone group and the TTM combined with the DC (TTM-DC) group.

### 2.2 Standard medical treatment and targeted temperature management

In our study, all patients were managed according to a standardized treatment protocol (6, 19, 20). Aneurysm clipping or coiling was performed within 24 h following emergency diagnostic evaluation using CTA or DSA. The primary objectives were to maintain a systolic blood pressure between 140 and 160 mmHg and to keep ICP < 20 mmHg before securing the aneurysm. After aneurysm clipping or coiling, a cerebral perfusion pressure (CPP) target of approximately 70 to 90 mmHg was established. Patients experiencing increased ICP were treated with a progressive, stepwise approach that included head elevation, ventilation control, sedation, analgesia, and hyperosmolar therapy. To manage refractory ICP, external ventricular drainage (EVD), lumbar drains (LD), 20% mannitol, hypertonic saline, furosemide, albumin, and DC were utilized. The procedure involved a large hemicraniectomy accompanied by duraplasty. A bone flap with a diameter of at least 12 cm was excised, encompassing the temporal, frontal, parietal, and portions of the occipital bones. Following this, the dura was opened, and a dural patch composed of a dura substitute was inserted into the incision and secured in place. Urapidil was utilized to manage acute hypertension following aSAH, while nimodipine was administered to prevent cerebral vasospasm. All included patients received DC during the TTM period in the TTM-DC group. TTM device (Temperature Management System 5,000, Arctic Sun, Medivance) used a temperature feedback system (TFS) and a medical-wrapped ice blanket (Arctic Gel Pads, Medivance) to provide a more rapid time to target temperature. TTM with core temperatures ranging from 36°C-37°C was performed immediately after poor-grade aSAH was diagnosed, together with emergency aneurysm repair. After aneurysm coiling or clipping, a rate of 1°C-2°C/h was used to rapidly reduce to 34°C-35°C. The TTM maintenance period was at least 72 h. The maximum temperature deviation for maintaining the target temperature was within 0.2°C-0.5°C. The core temperature for rewarming was 36°C-37.5°C, and the rewarming rate did not exceed 0.1°C-0.25°C/h. When reaching the target temperature of rewarming, continue to apply the normothermia mode of TFS for 48 h or until discharge. In the DC-alone group, the patient’s core temperature was maintained at 36°C-37°C. When the core temperature was 37°C-38.5°C, ice packs or an ordinary ice blanket were applied. When the temperature rose above 38.5°C, antipyretic and analgesic drugs (acetaminophen and indomethacin) were used to lower the temperature. Active anti-infective treatment was administered when infection was suspected. The details of the standard medical treatment and TTM protocol can be found in our previously published study (21).

### 2.3 Data collection

Patient data were collected prospectively using a case report form (CRF) at each hospital. The informed consent of patients’ relatives was obtained in this study. The baseline variables included age, sex, Hunt-Hess grade, mFisher grade, WFNS grade, GCS score, APACHE II score, location of the aneurysm, surgery methods, patients’ past medical history (including hypertension, coronary heart disease, diabetes, and stroke), smoking and drinking history, hydrocephalus, midline shift, initial temperature and the time operation from symptom onset. We used pooled names to represent anterior circulation and posterior circulation aneurysms. In this study, anterior circulation aneurysms included internal carotid artery (ICA), ophthalmic artery, anterior choroidal artery (AchA), posterior communicating artery (PComA), middle cerebral artery (MCA), anterior cerebral artery (ACA), and Anterior communicating artery (AComA). Posterior circulation aneurysms include basilar artery (BA), posterior cerebral artery (PCA), vertebral artery (VA), superior cerebellar artery (SCA), anterior inferior cerebellar artery (AICA), and posterior inferior cerebellar artery (PICA). Temperature data were obtained from the daily medical record system. Physiological parameters such as blood pressure, heart rate, and bladder temperature were collected at 2-h intervals. GCS scores, and laboratory data results including routine blood tests, liver and kidney function, serum electrolyte levels, blood glucose, arterial blood gas analysis, etc. were evaluated once a day. Relevant clinical data were collected by assessors excluded from the study. Head CT scans were repeated at 1, 3, and 7 days after disease onset. The assessment of outcomes was carried out by investigators who were blinded to the patient’s allocation and were not directly engaged in clinical decision-making. We also observed severe complications, including DCI, pneumonia, myocardial infarction, stress hyperglycemia, thrombocytopenia, acute liver injury, hypokalemia, and hypoproteinemia. These investigators contacted the patients or their family members via telephone or other means to complete the outcome assessment. The details of tests could be referred to our previously published study (21). DC was performed according to the decision of the officiating neurovascular neurosurgeon. Decision-making was based on clinical signs of herniation or brain swelling, the presence of large intracerebral hematoma (ICH), and/or intraoperative evidence of brain swelling.

### 2.4 Outcome measurement

The prognosis of neurological function 3 months after admission was assessed using the mRS score. The primary efficacy outcome was a favorable functional outcome defined as an mRS score of 0–3 at 90 days. Secondary outcomes were GCS score at discharge, proportion of EVD, proportion of tracheostomy, mechanical ventilation time, duration of stay in the ICU, length of hospitalization, DCI, midline shift, and hydrocephalus on the 7th day.

### 2.5 Statistical analysis

Data were reported as mean (± standard deviation, SD) or medians (interquartile ranges, IQR) or numbers (percentages, %) and compared using the t-test (normal distribution), Mann–Whitney U-test (no-normal distribution), or χ2 or Fisher’s exact test (categorical variables). Primary and secondary outcomes were compared by multivariable logistic regression analysis adjusted for age, sex, Hunt-Hess grade, mFisher grade, APACHE II score, baseline GCS score, location of aneurysm, surgery, hydrocephalus and midline shift at admission. In addition, after adjusting the above confounding factors, the GCS at discharge, duration of stay in the ICU, length of hospitalization, and mechanical ventilation time between TTM-DC and DC-alone groups were compared by linear regression analysis. Propensity score matching (PSM) analysis was performed to adjust for confounders and balance the observed covariates between the TTM-DC group and the DC-alone group. To reduce the impact of potential confounders, we employed 1:1 optimal pair matching. To demonstrate statistical accuracy, the unadjusted and adjusted odds ratios were provided with 95% confidence intervals. The aforementioned statistical analyses were performed using R software (version 4.2.2, R Core Team, Vienna, Austria), along with MSTATA software.^1^ The PSM analysis was performed using the R packages “MatchIt” and “optmatch.” Multivariable logistic regression and linear regression analysis used the “stats” package (glm and lm functions). Images were generated using the GraphPad Prism software (version 8, GraphPad Software, San Diego, CA, United States). Statistical significance was defined as a two-sided *p*-value <0.05.

## 3 Results

### 3.1 Patient characteristics

**Figure 1:**
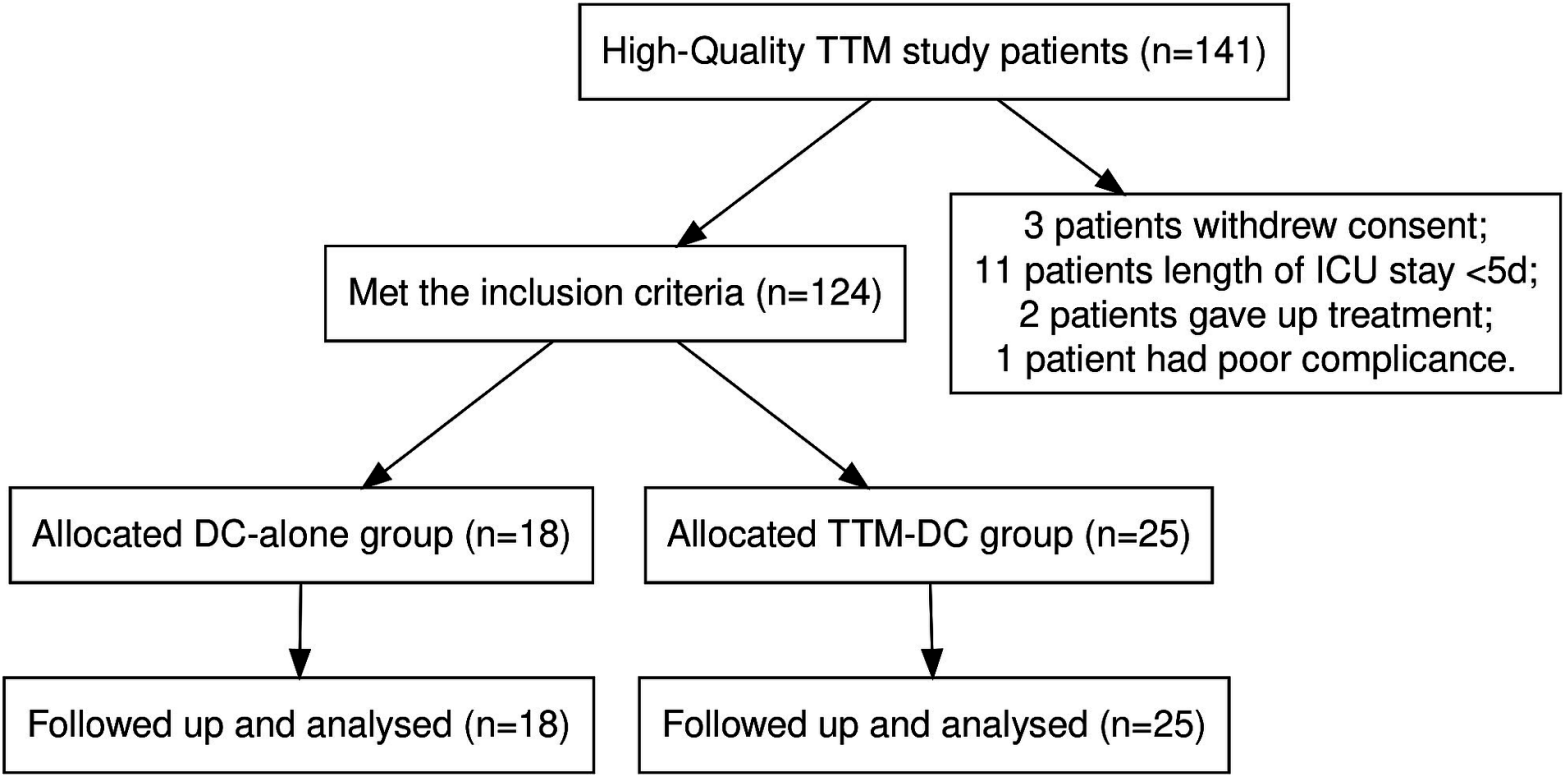
Flowchart of patients’ selection.

**Figure 2:**
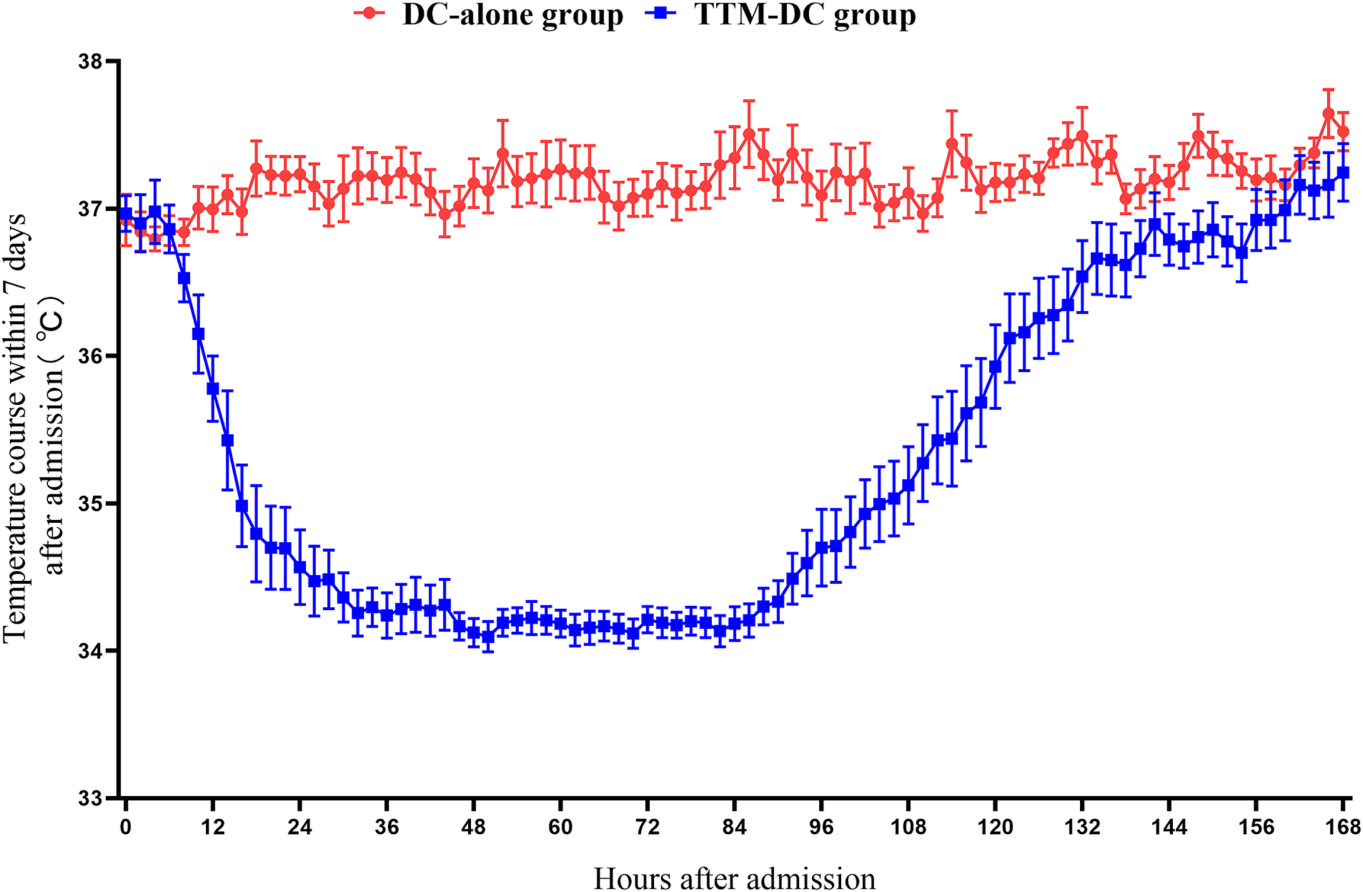
Core temperature for the first 7 days after admission. The core temperature of the patients in the DC-alone group was maintained at 36°C-37°C during the entire study period While the patients in the TTM-DC group maintained the target temperature of 34°C-35°C for at least 3 days Rewarming to 36°C within 01°C-025°C/h Normothermia control for at least 48 h Data were represented with mean and SE DC, decompressive craniectomy; TTM, targeted temperature management.

A total of 141 patients from three hospitals between April 2022 and April 2024 were included in the High-Quality TTM study. We excluded 17 patients according to the withdrawal criteria. Of these, three patients withdrew from the TTM during treatment, 11 patients due to length of ICU <5 days, one patient had poor compliance, and two patients abandoned treatment. 43 poor-grade patients were eligible for inclusion in this secondary analysis (Figure 1). There were 25 patients in the TTM-DC group and 18 patients in the DC-alone group. Sex, age, Hunt-Hess grade, WFNS score, mFisher grade, aneurysm location, surgical methods of aneurysm, GCS score, APACHE II score, initial temperature, time from onset to surgery, hydrocephalus, midline shift, hypertension, diabetes, coronary heart disease, and stroke history showed no baseline differences between groups. Baseline characteristics after propensity score matching are also shown in Table 1. The core temperature for the first 7 days after admission is shown in Figure 2.

**Table 1:** Baseline characteristic of patients with poor-grade aSAH.

Variables	Unmatched			Matched		
	TTM + DC, *N* = 25	DC, *N* = 18	*p*-value	TTM + DC, *N* = 18	DC, *N* = 18	*p*-value
Age, (year), Mean ± SD	60 ± 10	59 ± 12	0.661	60 ± 11	59 ± 12	0.768
Sex (Female: Male) [n (%)]	18:7	8:10	0.068	12:6	8:10	0.180
Hunt-Hess grade(IV:V)	20:5	17:1	0.375	14: 4	17:1	0.338
mFisher grade (3:4)	0:25	2:16	0.169	0:18	2:16	0.486
WFNS grade (IV:V)	22:3	14:4	0.427	17:1	14:4	0.338
Location, [n (%)]			0.252			0.229
Anterior circulation	22 (88.0%)	18 (100.0%)		15 (83.3%)	18 (100.0%)	
Posterior circulation	3 (12.0%)	0 (0.0%)		3 (16.7%)	0 (0.0%)	
Surgery, [n (%)]			0.766			>0.999
Clipping	17 (68.0%)	13 (72.2%)		13 (72.2%)	13 (72.2%)	
Coiling	8 (32.0%)	5 (27.8%)		5 (27.8%)	5 (27.8%)	
Smoking, [n (%)]	5 (20.0%)	5 (27.8%)	0.717	4 (22.2%)	5 (27.8%)	>0.999
Drinking, [n (%)]	4 (16.0%)	5 (27.8%)	0.455	3 (16.7%)	5 (27.8%)	0.691
Hypertension, [n (%)]	16 (64.0%)	7 (38.9%)	0.103	10 (55.6%)	7 (38.9%)	0.317
Diabetes, [n (%)]	1 (4.0%)	1 (5.6%)	>0.999	1 (5.6%)	1 (5.6%)	>0.999
Coronary heart disease, [n (%)]	3 (12.0%)	4 (22.2%)	0.427	2 (11.1%)	4 (22.2%)	0.658
History of stroke, [n (%)]	6 (24.0%)	9 (50.0%)	0.078	5 (27.8%)	9 (50.0%)	0.171
Hydrocephalus, [n (%)]	8 (32.0%)	7 (38.9%)	0.640	4 (22.2%)	7 (38.9%)	0.278
Midline shift, [n (%)]	12 (48.0%)	13 (72.2%)	0.112	9 (50.0%)	13 (72.2%)	0.171
APACHE II, Mean ± SD	21.7 ± 3.8	21.6 ± 3.6	0.885	21.6 ± 4.1	21.6 ± 3.6	>0.999
GCS, Median (IQR)	4.00 (3.00, 5.00)	5.00 (3.00, 6.00)	0.282	3.50 (3.00, 5.00)	5.00 (3.00, 6.00)	0.136
Initial temperature, (°C), Median (IQR)	36.50 (36.50, 36.80)	36.65 (36.53, 36.90)	0.204	36.50 (36.33, 36.80)	36.65 (36.53, 36.90)	0.293
Time from hemorrhage to operation Median (IQR)	12.0 (10.0, 15.8)	13.0 (10.8, 18.0)	0.103	11.5(10.0,16.6)	13.0 (10.8, 18.0)	0.668

APACHE II, Acute Physiology and Chronic Health Evaluation II; GCS, Glasgow Coma Scale; mFisher, Modified Fisher Scale; WFNS, World Federation of Neurosurgical Societies. Values are expressed as median (IQR), number (%), or mean ± standard deviation (SD). Statistical methods: Welch Two Sample t-test; Wilcoxon rank sum test; Pearson’s Chi-squared test; Fisher’s exact test.

### 3.2 Outcomes

**Figure 3:**
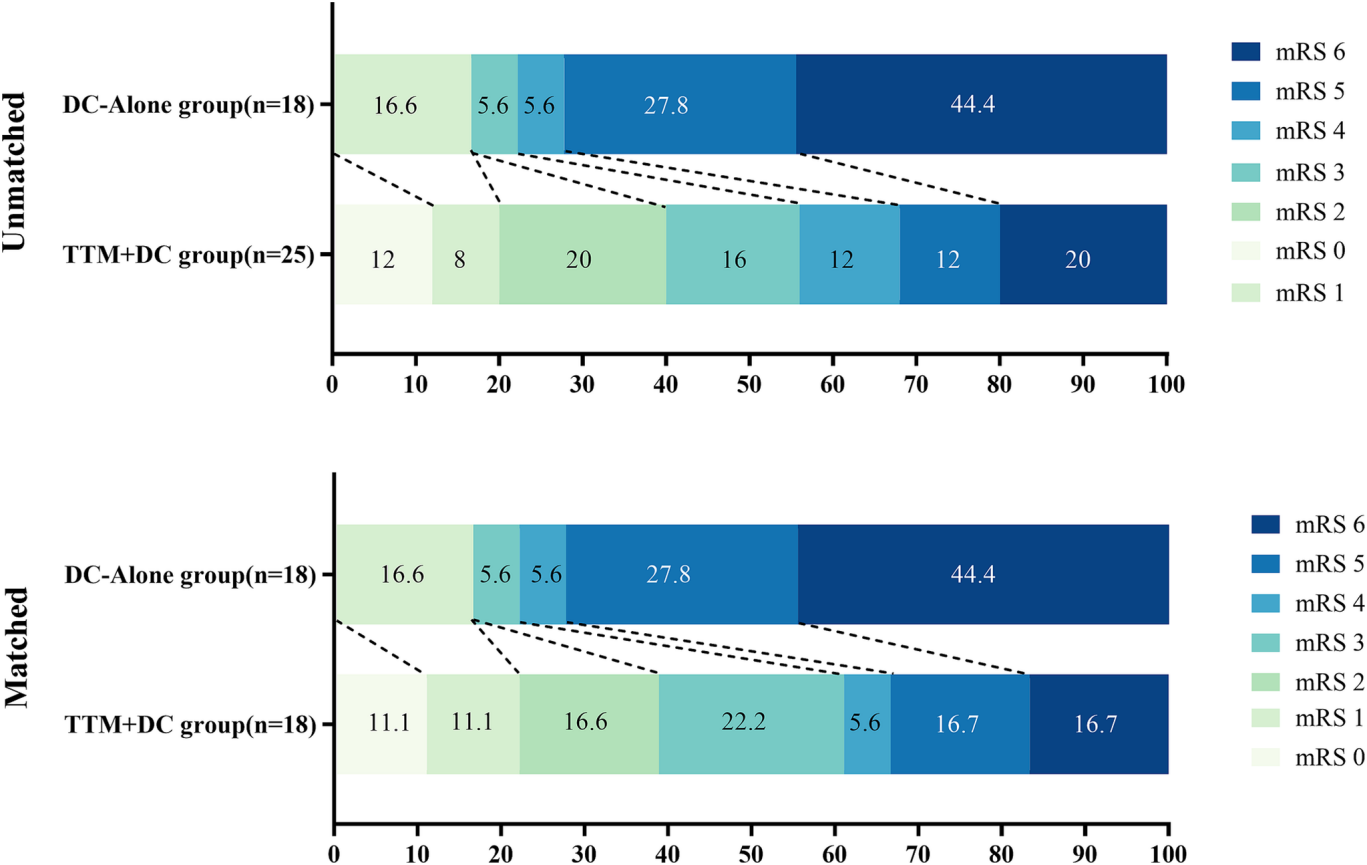
The distribution of mRS scores at 90 days favored TTM-DC. The mRS scores ranged from 0 to 6, with higher scores indicating greater disability and 6 indicating death DC, decompressive craniectomy; TTM, targeted temperature management.

The rate of favorable outcomes in the TTM-DC group was higher than that in the DC-alone group (56% vs. 22%, aOR 5.97, 95%CI: 0.96–52.2, *p* = 0.071) at 3-month, but the difference was not significant. After propensity score matching analysis, the rate of favorable outcome was significantly higher in the TTM-DC group than in the DC-alone group (61% vs. 22%, OR 5.50, 95%CI: 1.36–26.3, *p* = 0.022). After adjusting for age, sex, Hunt-Hess grade, mFisher grade, APACHE II score, location of the aneurysm, surgery, hydrocephalus, and midline shift at admission, the between-group difference in GCS at discharge (9 vs. 3, *β* 2.58, 95%CI:0.32–4.84, *p* = 0.032) significantly favored the TTM-DC group. In addition, the proportion of midline shift on the 7th day was lower in the TTM-DC group than in the DC-alone group (36% vs. 67%, aOR 0.15, 95%CI:0.02–1.13, *p* = 0.065), but the difference was not significant. After propensity matching analysis, the proportion of midline shift on the 7th day was significantly lower in the TTM-DC group than in the DC-alone group (33% vs.67%, OR 0.25, 95%CI: 0.06–0.96, *p* = 0.050). There were no group differences in the occurrence rate of DCI, proportion of EVD, hydrocephalus, tracheostomy, mechanical ventilation time, length of hospitalization, and duration of stay in the ICU. The distribution of mRS scores on the 90th day for both groups is summarized in Figure 3 (Table 2).

**Table 2:** Efficacy outcomes in patients with poor-grade aSAH.

	Overall analysis					Propensity score matching				
Variables	TTM + DC group	DC-Along group	Effect variable	Adjusted value (95% CI)	*p*	TTM + DC group	DC-Along group	Effect variable	Unadjusted value (95% CI)	*p*
mRS 0–3, [n (%)]	14 (56%)	4 (22%)	OR	5.97(0.96, 52.2)	0.071	11 (61%)	4 (22%)	OR	5.50(1.36, 26.3)	**0.022**^**a**^
GCS at discharge, Median (IQR)	9.0 (3.0, 12.0)	3.0 (3.0, 6.75)	Beta	2.58(0.32, 4.84)	**0.032**^**a**^	8.5 (5.0, 11.25)	3.0 (3.0,6.75)	Beta	2.72(0.59–4.85)	**0.017**^**a**^
Duration of stay in the ICU (days), Median (IQR)	23 (12, 31)	13 (5, 20)	Beta	7.95(−1.76, 17.67)	0.118	21 (11, 29)	13 (5, 20)	Beta	6.94(1.26,15.15)	0.106
Length of hospitalization(days), Median (IQR)	23 (12, 31)	17 (6, 27)	Beta	4.78(−5.86, 15.42)	0.385	23 (13, 29)	17 (6, 27)	Beta	4.22(−4.69,13.13)	0.360
Mechanical ventilation time, (days), Median (IQR)	19 (11, 31)	14 (7, 21)	Beta	7.32(−2.77, 17.41)	0.165	14 (10, 29)	14 (7, 21)	Beta	4.50(−3.89,12.89)	0.301
Tracheostomy, [n (%)]	18 (72%)	9 (50%)	OR	4.36(0.65, 39.6)	0.153	13 (72%)	9 (50%)	OR	2.60 (0.67, 11.05)	0.176
EVD, [n (%)]	6 (24%)	2 (11%)	OR	4.48(0.32, 230)	0.334	6 (23%)	2 (11%)	OR	4.00(0.77, 30.74)	0.124
Midline shift, [n (%)]	9 (36%)	12 (67%)	OR	0.15(0.02, 1.13)	0.065	6 (33%)	12 (67%)	OR	0.25 (0.06, 0.96)	**0.050**^**a**^
Hydrocephalus, [n (%)]	7 (28%)	7 (39%)	OR	0.26(0.02, 3.64)	0.319	4 (22%)	7 (39%)	OR	2.23(0.53, 10.4)	0.280
DCI, [n (%)]	10 (40%)	10 (56%)	OR	0.64(0.12, 3.35)	0.598	8 (44%)	10 (56%)	OR	1.56(0.42, 5.98)	0.510

OR, Odds Ratio; CI, Confidence Interval. *Adjusted for Age, Sex, Hunt-Hess grade, mFisher grade, APACHE II score, location of the aneurysm, Surgery, Hydrocephalus at admission, and Midline Shift at admission. Variables are expressed as mean ± SD and median (IQR) for continuous variables or number (%) for categorical variables. DCI, delayed cerebral ischemia; DC, decompressive craniectomy; EVD, external ventricular drainage; ICU, Intensive Care Unit; mRS, Modified Rankin Scale; GCS, Glasgow Coma Scale. TTM, targeted temperature management.

a Significant differences between groups. The bold values provided mean *p* < 0.05.

### 3.3 Safety

In the TTM-DC group, five patients (20%) died, compared to eight patients (44%) in the DC-alone group (OR 0.31, 95% CI: 0.08–1.21, *p* = 0.091). Pneumonia occurred in 13 patients (52%) in the TTM-DC group and 11 patients (61%) in the DC-alone group (OR 0.69, 95% CI: 0.20–2.36, *p* = 0.553). Myocardial infarction occurred in eight patients (32%) in the TTM-DC group and six patients (33%) in the DC-alone group (OR 0.94, 95% CI: 0.26–3.42, *p* = 0.927). Stress hyperglycemia was present in 11 patients (44%) in the TTM-DC group and 11 patients (61%) in the DC-alone group (OR 0.50, 95% CI: 0.15–1.72, *p* = 0.271). Thrombocytopenia occurred in three patients (12%) in the interrupted TTM-DC group and one patient (5.6%) in the DC-alone group (OR 2.32, 95%CI: 0.22–24.31, *p* = 0.483). Acute liver injury occurred in four patients (16%) in the interrupted TTM-DC group and five patients (28%) in the DC-alone group (OR 0.50, 95% CI: 0.11–2.19, *p* = 0.354). Hypokalemia was observed in eight patients (32%) in the interrupted TTM-DC group and six (33%) in the DC-alone group (OR 0.94, 95%CI: 0.26–3.42, *p* = 0.927). Hypoproteinemia occurred in 10 patients (40%) in the TTM-DC group and seven patients (39%) in the DC-alone group (OR 1.05, 95%CI: 0.30–3.62, *p* = 0.941). After adjusting for age, sex, Hunt-Hess grade, mFisher grade, APACHE II score, location of the aneurysm, surgery, hydrocephalus at admission, and midline shift at admission, the above complications had no significant difference between both groups (Table 3).

**Table 3:** Safety outcomes in patients with poor-grade aSAH.

Variables	TTM + DC group	DC-alone group	Unadjusted value OR (95%CI)	*p*	Adjusted value OR (95%CI)	*p*
Mortality, [n (%)]	5 (20%)	8 (44%)	0.31 (0.08, 1.21)	0.091	0.47 (0.08, 2.64)	0.392
Pneumonia, [n (%)]	13 (52%)	11 (61%)	0.69 (0.20, 2.36)	0.553	0.80 (0.17, 3.69)	0.776
Myocardial infarction, [n (%)]	8 (32%)	6 (33%)	0.94 (0.26, 3.42)	0.927	0.78 (0.14, 4.39)	0.779
Stress hyperglycemia, [n (%)]	11 (44%)	11 (61%)	0.50 (0.15, 1.72)	0.271	0.21 (0.03, 1.73)	0.147
Thrombocytopenia, [n (%)]	3 (12%)	1 (5.6%)	2.32 (0.22, 24.31)	0.483	35.87 (0.14, 90.95)	0.205
Acute liver injury, [n (%)]	4 (16%)	5 (28%)	0.50 (0.11, 2.19)	0.354	0.32 (0.04, 2.55)	0.284
Hypokalemia, [n (%)]	8 (32%)	6 (33%)	0.94 (0.26, 3.42)	0.927	0.93 (0.15, 5.71)	0.941
Hypoproteinemia, [n (%)]	10 (40%)	7 (39%)	1.05 (0.30, 3.62)	0.941	0.71 (0.13, 3.70)	0.681

*Adjusted for Age, Sex, Hunt-Hess grade, mFisher grade, APACHE II score, location of the aneurysm, Surgery, Hydrocephalus at admission, and Midline Shift at admission. Variables are expressed as mean ± SD and median (IQR) for continuous variables or number (%) for categorical variables. DCI, delayed cerebral ischemia; DC, decompressive craniectomy; TTM, targeted temperature management.

## 4 Discussion

This study evaluated the impact of TTM combined with DC on outcomes in patients with poor-grade aSAH, utilizing prospective, multicenter data. We found that TTM-DC was associated with improved neurological functional outcomes at 90 days. Additionally, TTM-DC was shown to increase the GCS score of patients at discharge. This finding was further supported by 1:1 propensity score matching analyses. Furthermore, TTM-DC was associated with a reduction in the midline shift rate by the 7th day after adjusting several covariates, although this difference was not statistically significant. However, propensity score matching confirmed that TTM-DC could reduce the midline shift rate compared to the DC-alone group. In terms of secondary clinical outcomes, the DCI, duration of ICU stay, mechanical ventilation time, length of hospitalization, rates of tracheostomy, hydrocephalus on the 7th day, and 90-day mortality did not differ between the TTM-DC group and the DC-alone group. Importantly, the risk of safety outcomes associated with TTM-DC, such as pneumonia, myocardial infarction, stress hyperglycemia, thrombocytopenia, acute liver injury, hypoproteinemia, and hypokalemia, did not increase compared to the DC-alone group.

The above results of hypothermia treatment indicated that the TTM procedure of poor-grade aSAH requires further improvement and optimization. Our previous pilot study demonstrated that a high-quality TTM regimen significantly enhanced neurological outcomes at 3 months (46.7% vs. 20.0%, *p* = 0.028) (21). DC is a life-saving management technique employed in patients with poor-grade aSAH in the EBI stage. Although evidence supporting the routine use of DC for the management of elevated ICP in poor-grade aSAH is limited, it is considered a last-resort option when medical management proves ineffective. Nonetheless, the effects of DC on functional outcomes and mortality remain uncertain. Smith et al. suggested the DC has potential advantages on local and global ICP control for the treatment of poor-grade aSAH due to improved brain perfusion. Eight patients experienced the DC and five patients had a remarkably favorable outcome (22). D’Ambrosio et al. found that poor-grade aSAH patients associated with ICH undergoing DC had a lower overall mortality rate (25% vs. 40%) compared with the control group at 3 months. However, both groups were similar in 12-month mortality rates (42% vs. 40%). Although this intervention may decrease mortality, the quality of life (QoL) for those who survive using this procedure was poor (23). Simon et al. conducted a single-center cohort and performed a systematic review of the literature to analyze the effect of DC on long-term clinical outcomes in poor-grade aSAH patients. Patients in the DC group had a significantly worse clinical status at presentation. The incidence of additional ICH occurred in the DC group was higher than that in the control group (68% vs.27%, *p* = 0.0002). Favorable outcomes (20% vs. 20%, *p* = 0.61) and mortality rate (55% vs. 51%) after 2 years did not differ between the two groups. Nine eligible studies were comprehensively analyzed, and it was found that nearly half of the patients (49%) achieved good long-term functional outcomes, and the total mortality rate was 24% (24). A matched control study of Hai et al. published poor-grade patients who had DC experienced a statistically significant decrease in short-term mortality compared with those without DC (23.8% vs. 41.7%, *p* < 0.05) and showed a decrease in intracranial pressure peaked (22.25 ± 4.24 mmHg, *p* < 0.001) after surgery. However, there was no significant difference in the long-term favorable outcomes (3-year GOS 4–5:33.4% vs. 37.5%) between the two groups (25). Therefore, DC can bring some benefits to patients with poor-grade aSAH, but other methods are still needed to assist treatment.

The therapeutic effect of DC combined with TTM on various neurological diseases has not yielded consistently positive results in other studies. A multicenter randomized controlled trial by Hui et al. found no significant difference in ICP values between the normothermia and hypothermia groups, as well as their respective subgroups with or without DC (*p* = 0.45) (26). In addition, in Fan et al.’s randomized controlled trial, the DC plus head surface cooling group seemed to have a higher proportion of good outcomes, but there was no significant difference between the groups (*p* = 0.598) (27). There is currently a lack of relevant analysis regarding the combination of TTM and DC in patients with poor-grade aSAH. Our secondary analysis of a prospective multicenter study on high-quality TTM suggests that TTM combined with DC in this study may be a promising treatment modalities. Prior to the analysis of PSM, we observed lower rates of unfavorable outcomes (44% vs. 78%, *p* = 0.071) and mortality (20% vs. 44%, *p* = 0.091) in the TTM-DC group compared to the DC-alone group at 3 months; however, these differences did not reach statistical significance. Several studies have indicated that TTM may result in various complications, including heart disease, hypotension, coagulation dysfunction, infections, shivering, hyperglycemia, electrolyte imbalances, and gastrointestinal dysfunction (28–31). The potential complications and significant risks associated with therapeutic hypothermia cannot be ignored. They concluded that TTM should be employed with caution as a standard treatment option for patients with poor-grade aSAH.

There were no significant differences in complications between the DC-alone group and the TTM-DC group; however, we should remain cautious regarding the high incidence of related infections, such as pneumonia. We observed that the incidence of thrombocytopenia in the TTM-DC group was higher than that in the DC-alone group, and hypothermia may influence coagulation function. Therefore, to mitigate the high incidence of other complications, it is crucial to closely monitor patients and implement appropriate countermeasures (32, 33). The TTM protocol was initiated immediately upon the patient’s admission to ensure optimal brain protection. The observed reduction in the rate of midline shift is encouraging, suggesting a gradual recovery of brain compliance. However, it is important to note that this finding may also be affected by limitations in sample size.

Our findings provide preliminary data suggesting that such trials may be beneficial. Additionally, we have several questions that need to be considered: (1) What is the overall efficacy of TTM in patients with poor-grade aSAH? (2) When should TTM be initiated, and for how long should hypothermia be maintained? (3) Should TTM serve as a foundation for the early implementation of DC, or should it be initiated only when DC is not yielding satisfactory results? Throughout the hospital stay, TTM was implemented with the following strategy in our study: normothermia control upon admission using TFS, rapid attainment of the target temperature range of 34°C to 35°C following aneurysm surgery, avoidance of temperature fluctuations during the maintenance phase, and gradual rewarming until a normal temperature was sustained for at least 48 h.

This study has several limitations that warrant cautious interpretation of our findings. As a multicenter non-randomized controlled trial, our analysis was restricted to patients receiving DC treatment, which may introduce selection bias. Additionally, the relatively small sample size limits our ability to draw definitive conclusions regarding the differences between the two groups. Consequently, this study employed propensity score matching to mitigate potential bias between the two groups. We plan to expand the sample size in future research to achieve more accurate results. This study is limited to prognostic assessment after a three-month period, and the short-term clinical outcomes may not accurately reflect the long-term effects of treatment. Therefore, we will continue to follow up with patients to evaluate their long-term prognosis and survival status. Furthermore, the absence of available ICP data restricted a more comprehensive discussion on neurological monitoring. In subsequent studies, we will continue to record ICP data throughout the trial. In follow-up studies, we intend to conduct a multicenter randomized controlled trial of TTM combined with DC intervention, guided by multimodal neurofunctional monitoring, to further validate our findings.

## 5 Conclusion

TTM combined with DC can improve short-term neurological outcomes and ameliorate clinical conditions at discharge in poor-grade aSAH patients. TTM should be considered one of the main treatments for poor-grade aSAH patients who underwent DC.

## Data Availability

The raw data supporting the conclusions of this article will be made available by the authors, without undue reservation.

## Footnotes

^1^ www.mstata.com
